# Different chromatin and energy/redox responses of mouse morulae and blastocysts to slow freezing and vitrification

**DOI:** 10.1186/s12958-015-0018-z

**Published:** 2015-03-24

**Authors:** Bence Somoskoi, Nicola A Martino, Rosa A Cardone, Giovanni M Lacalandra, Maria E Dell’Aquila, Sandor Cseh

**Affiliations:** Department and Clinic of Obstetrics and Reproduction, Szent Istvan University, Budapest, Hungary; Veterinary Clinics and Animal Productions Unit, Department of Emergency and Organ Trasplantation (DETO), University of Bari Aldo Moro Valenzano, Bari, Italy; Department of Bioscience, Biotechnology and Pharmacological Science, University of Bari, 70126 Bari, Italy

**Keywords:** Mouse embryos, Slow freezing, Vitrification, Nuclear chromatin, Mitochondria, Reactive oxygen species

## Abstract

**Background:**

The ability to cryopreserve mammalian embryos has become an integral part of assisted reproduction, both in human and veterinary medicine. Despite differences in the size and physiological characteristics of embryos from various species, the embryos have been frozen by either of two procedures: slow freezing or vitrification. The aim of our study was to compare the effect of slow freezing and vitrification to the chromatin structure, energy status and reactive oxygen species production of mouse morulae and blastocysts.

**Methods:**

Mouse morulae and blastocysts were randomly allocated into vitrification, slow freezing and control groups. For slow freezing, Dulbecco phosphate buffered saline based 10% glicerol solution was used. For vitrification, G-MOPS™ based solution supplemented with 16% ethylene glycol, 16% propylene glycol, Ficoll (10 mg/ml) and sucrose (0.65 mol/l) was used. After warming, the chromatin integrity, mitochondrial distribution pattern and energy/oxidative status were compared among groups.

**Results:**

Cryopreservation affected chromatin integrity at a greater extent at the morula than the blastocyst stage. Chromatin damage induced by slow freezing was more relevant compared to vitrification. Slow freezing and vitrification similarly affected mitochondrial distribution pattern. Greater damage was observed at the morula stage and it was associated with embryo grade. Cryopreservation altered the quantitative bioenergy/redox parameters at a greater extent in the morulae than in the blastocysts. Effects induced by slow freezing were not related to embryo grade or mitochondrial pattern, as affected embryos were of all grades and with both mitochondrial patterns. However, effects induced by vitrification were related to mitochondrial pattern, as only embryos with homogeneous mitochondrial pattern in small aggregates had reduced energy status.

**Conclusions:**

This study shows for the first time the joint assessment of chromatin damage and mitochondrial energy/redox potential in fresh and frozen mouse embryos at the morula and blastocyst stage, allowing the comparison of the effects of the two most commonly used cryopreservation procedures.

## Background

The ability to cryopreserve mammalian embryos has become an integral part of assisted reproductive technologies (ART) in both human and veterinary medicine. Despite differences in the size and physiological characteristics of embryos from various species, most embryos have been frozen by either one of two procedures: the traditional slow (equilibrium) cooling and freezing method of cryopreservation (CP), namely slow freezing (SF), and the rapid procedure (non-equilibrium cooling) referred to as vitrification (VF) [1,2]. The same fundamental cryobiological principles operate to determine the survival of embryos cryopreserved by both methods [3-5]. In order to avoid the so-called freezing damage during CP of embryos, the freezing solution is supplemented with cryoprotective additives (CPAs). There are differences in the permeability to permeating CPAs and sensitivity to cooling among the embryos of different species. Furthermore, the earlier the stages of development, the less permeable are the embryos and the survival rates of cryopreserved embryos increases as the developmental stage proceeded [1,6,7]. This higher cryotolerance of late stage embryos is the outcome of higher nucleus-cytoplasm ratio [8]. At the traditional SF, the progressive dehydration of the embryo is based on 1) the equilibration of embryos prior to cooling in freezing solution supplemented with low concentration of CPA (1–2 M) and 2) slow cooling to minus 30-40°C, before plunging into liquid nitrogen for long term storage at minus 196° C. Vitrification is an alternative approach for cryopreservation, which avoids the formation of ice crystals in the intracellular and extracellular space [9,10,2]. This process is achieved by a combination of a very high concentration of CPAs (4–8 M, a solution with very high viscosity) and an extremely high cooling rate which result in the solidification of the solution without ice crystal formation (the solution vitrifies) [3,11].

Appropriate mitochondrial (mt) distribution and membrane potential in embryos are very important for their developmental potential and for a variety of cellular activities, including ATP synthesis and specific cell functions [12,13]. However, there is little information available about the effects of CP (traditional SF and VF) on mt dynamics/distribution and reactive oxygen species (ROS) production in embryos. In an early study, Noto et al. [14] found that rapid freezing did not affect subcellular structures. The well organized and specific mt distribution appeared still to be present after frozen storage and subcellular structures seemed to be rather resistant targets for cryoinjury [14]. Zhao et al. [15] found that the mt ring rate decreased in mouse 2-PN (fertilized egg) embryos after VF, an event which may affect the subsequent developmental viability of the embryos. Similar results were found by Shi et al. [16] indicating that VF alters mt distribution in porcine metaphase II (MII, matured) oocytes. Tanim et al. [17] analyzed the impact of different factors on the outcome of human ART. Their data indicate that among other things, gamete/embryo CP may be associated with mitochondria, genetic and epigenetic alterations to gametes/embryos [17]. A recent study shows remarkable decrease in mt activity in slow frozen and vitrified sheep embryos compared with fresh ones [18].

Information about the effects of CP on chromatin integrity are incomplete and contradictory. Kader et al. [19] investigated the chromatin integrity index in fresh, vitrified and slow-frozen blastocysts and found significant decrease in vitrified embryos. However, Li et al. [20] found higher DNA integrity in vitrified human and mouse blastocysts than in the slow-frozen ones and similar to fresh blastocysts. Isachenko et al. [21] investigated the effect of integrity rate of pronuclei after the CP of pronuclear-zygotes on subsequent embryo development and pregnancy. Their observation indicates that it is a predictor of future embryo development and implantation. The high integrity rate resulted in high pregnancy rate, while zygotes with low integrity of pronuclei after CP had low developmental potential [21].

It has been reported that oxidative stress (OS) may be an important mechanism underlying the toxic effects of CP procedures which then may trigger the apoptotic cascade leading to a decrease in the survival rate and developmental rate of gametes/embryos after thawing [22-25]. Oxidative stress occurs if disequilibrium takes place between ROS production and antioxidative capacity of the cell [26] and it has also been implicated in the etiology of some forms of female infertility [27]. Mitochondria represent the major source of ROS, in which they are produced in a stepwise process [28]. Under physiological conditions, ROS are neutralized by an elaborate defense system consisting of enzymes (e.g. catalase, superoxide dismutase, glutathione peroxidase or reductase) and non enzymatic antioxidants (e.g. vitamin C, E, A, pyruvate, glutathione, ubiquinone, taurine, hypotaurine) [29]. Thus, any perturbation in mt activity or in the activity of scavenger systems can lead to profound implications in ROS production, OS induction, and mt cytochrome c release, which is an important step for apoptosis [30].

Embryos, as other aerobic cells, produce ATP and ROS by means of mt oxidative phosphorylation. Blastocyst freezing was abandoned for years, since only 25% of the zygotes were able to reach the blastocyst stage in vitro in usual culture media and low pregnancy rates were reported. Recently the situation has been changed, because the improved embryo culture systems, the newly developed sequential media, and furthermore the improved CP procedures have been increased the outcome of in vitro blastocyst production, the survival of cryopreserved blastocysts and the pregnancy rates of frozen-thawed IVF cycles [31]. According to the International Embryo Transfer Society (IETS) the total number of transferred embryos, produced in vitro or in vivo, for cattle and small ruminant species were approximately 800.000 and 2.000, respectively (data recorded in 2009) [32]. The best stages for CP and transfer of frozen cattle embryos are compact morulae/early blastocysts at Day 6 (D6, when IVF is on D0) and expanded blastocysts (D7-8). Most of the assessment for either cryopreservation or fresh transfer is at D7-8. To our knowledge, no comparative research regarding the effect of SF and VF on mitochondrial distribution, chromatin integrity, energy status and ROS production in different developmental stages (mouse morulae and blastocysts) of embryos has been documented.

Therefore, the aim of the present study was to investigate and compare the effects of the traditional controlled SF and VF on chromatin integrity, mt distribution, energy status and intracellular levels of ROS in mouse embryos being in morula and blastocyst stages.

## Methods

### Embryo recovery and in vitro culture

Procedures with animals were performed following good veterinary practice for animal welfare according to Hungarian national laws in force. The protocol of the animal experiment was approved by the Ethical Committee of the Faculty of Veterinary Science Szent Istvàn of Budapest. Embryos were produced as reported by Klambauer et al. [33]. Briefly, eight weeks old CB6F1 female mice (Institute of Oncology, Animal Care Facility, Budapest, Hungary) were superovulated by 10 IU eCG i.p., followed by 10 IU hCG i.p. 48 h later, in order to induce the final maturation of the oocytes and ovulation. After the hCG injection the females were paired with males (1 female/male), then 20 to 24 h later the embryos were collected (Day 1). Until CP (with SF or VF) or analysis (fresh control embryos), the embryos were cultured in G1 medium (Vitrolife, Goteborg, Sweden) at 37.5°C with 6.5% CO_2_ and maximal humidity in air for further 96 h. Only morphologically normal embryos, being at the compact morula or blastocyst stage, were randomly destined to either SF or VF or fresh control groups. Five replicates were performed in all treatments.

### Slow programmable freezing

After equilibration in a medium containing 10% glycerol, Dulbecco phosphate bufferd saline (DPBS + 10% FCS + 10% glycerol) for 10–15 min, the embryos were sucked up into straws (5 embryo per straw) [34-37]. The straws containing the embryos were then transferred into a Planer freezing machine (PLANER R 205, Planer, Sunbury-on-Thames, Middlesex UK) pre-cooled to minus 7°C. After a 10 min waiting period, the samples were allowed to cool down to minus 7°C. Once reached this temperature, artificial induction of the ice formation with a pre-cooled forceps was performed (seeding). After 10 min waiting, cooling down the embryos to minus 33°C was performed with cooling speed 0.3°C/minute. Finally, the embryos were transferred into liquid nitrogen (LN_2_) and stored for one week. Thawing was performed by keeping the straw in air for 20 sec (air thaw), followed by 30 sec in warm water (25°C). Cryoprotectant (CPA) was removed f rom the embryos in 4 steps: 5 min in medium containing 6% glycerol + 0.3 M sucrose; 5 min in medium containing 3% glycerol + 0.3 M sucrose; 5 min in medium containing 0.3 M sucrose and, finally, 5 min in PBS + 20% FCS.

### Vitrification

Embryo VF was performed with the VitroLoop VF procedure as previously described [33] and unless otherwise specified, all materials were provided by Vitrolife. Briefly, embryos were exposed to a 2-step loading of the CPA solution, ethylene glycol (EG) and propylene glycol (PG), before being placed on a thin filmy layer formed from the VF solution in a small nylon loop, then they were rapidly submerged in liquid nitrogen (LN_2_) and stored for one week. VF was carried out in RapidVit Cleave VF solutions (Vitrolife; solution 1: holding medium, solution 2: equilibration medium, and solution 3: VF medium) and embryos were manipulated in 4-well culture dish (Nunc Intermed, Roskilde, Denmark) held on a warming plate at 37°C. The holding or basic solution is based on G-MOPS™ and was supplemented with gentamycin and human serum albumin (HSA, 5 μg/ml). Both the equilibration and the VF media are based on the holding/basic solution, but the equilibration medium was supplemented with 8% EG + 8% PG and the VF solution was enriched with 16% EG, 16% PG, F (Ficoll, F-400, 10 mg/ml) and S (Sucrose, 0.65 mol/l). All manipulations of the embryos during their preparation for VF were carried out at 37°C (on a heated stage). Embryos were suspended from solution 1 into the equilibration medium (solution 2) for 2 min. Thereafter, they were transferred and washed quickly in small drops of VF medium (solution 3). The cryoloop was dipped into the VF medium to create a thin filmy layer of the solution on the nylon loop where embryos (max 3 embryos) were quickly transferred from the VF medium. Within 30 sec of suspension in the VF medium, the loop with the embryos was plunged into LN2. Embryos were warmed and rehydrated by a 3-step dilution of the CPA performed at 37°C. At warming, the embryos were moved through a series of G-MOPSTM solutions containing the S in decreasing concentrations (warming solution 1: 0.65 mol/l; 30 sec, warming solution 2: 0.25 mol/l; 1 min, warming solution 3: 0.125 mol/l; 2 min and warming solution 4: 0.0 mol/l; 5 min) (RapidWarm Cleave; Vitrolife, Goteborg, Sweden).

### Mitochondria and ROS staining

Embryos underwent mitochondria/ROS staining following the procedure by Ambruosi et al. [38], Martino et al. [39] and Martino et al. [52]. Embryos were washed three times in PBS with 3% bovine serum albumin (BSA) and incubated for 30 min in the same medium containing 280 nM MitoTracker Orange CMTM Ros (Molecular Probes M-7510, Oregon, USA) at 38.5°C under 5% CO2 in air. The cell-permeant probe contains a thiol-reactive chloromethyl moiety. Once the MitoTracker probe accumulates in the mitochondria, it can react with accessible thiol groups on peptides and proteins to form an aldehyde-fixable conjugate. This cell-permeant probe is readily sequestered only by actively respiring organelles depending on their oxidative activity [40,41]. After incubation with MitoTracker Orange CMTM Ros, embryos were washed three times in PBS with 0.3% BSA and incubated for 15 min in the same media containing 10 mM 2’,7’-dichlorodihydrofluorescein diacetate (DCDHF DA). The non-ionized DCDHF DA is membrane permeant and therefore is able to diffuse readily into cells. Once within the cell, the acetate groups are hydrolysed by intracellular esterase activity forming 2’,7’-dichlorodihydrofluorescein (DCDHF) which is polar and thus trapped within the cell. DCHF fluoresces when it is oxidized by H_2_O_2_ or lipid peroxides to yield 2’,7’-dichlorofluorescein (DCF). The level of DCF produced within the cells is linearly related to that of peroxides present and thus its fluorescent emission provides a measure of the peroxide levels [42]. After incubation, embryos were washed three times in pre-warmed PBS without BSA and fixed with 3.7% paraformaldehyde solution in PBS. All procedures after thawing/warming were performed within 1 hour. Embryos were kept in fixative at 4°C for no longer than two to three days. The organelle-specificity of the mt probe was assessed, as reported by Valentini et al. [43], in control samples which were imaged after incubation in MitoTracker Orange and further incubation for 5 min in the presence of 5 mM of the mt membrane potential (Delta Psi)-collapsing uncoupler carbonyl cyanide 3-chloro phenyl hydrazone (CCCP; Molecular Probes), which inhibits mt respiratory activity thus reducing fluorescence intensity. Particular attention was paid to avoid sample exposure to the light during staining and fixing procedures in order to reduce photobleaching.

### Detection of the nuclear chromatin status

To evaluate nuclear chromatin, embryos were stained with 2.5 μg/ml Hoechst 33258 in 3:1 (v/v) glycerol/PBS, mounted on microscope slides, covered with cover-up micro slides, sealed with nail polish and kept at 4°C in the dark until observation [38]. Nuclear chromatin status was observed un der a Nikon Eclipse 600 fluorescent microscope equipped with B2A (346 nm excitation/ 460 nm emission) filter. Embryos were classified as normal (grade A) when the presence of a regular-shaped nucleus inside each blastomere was observed. The formation of micronuclei and lobulated nuclei was considered as signs of chromatin damage [44]. Embryos showing 0 to 20% affected blastomeres were classified as grade B and embryos with more than 20% affected blastomeres were classified as grade C.

### Mitochondrial distribution pattern and intracellular ROS localization

For mt distribution pattern evaluation, embryos were observed at 600 x magnification in oil immersion with a Nikon C1/TE2000-U laser scanning confocal microscope. A helium/neon laser ray at 543 nm and the G-2 A filter (551 nm exposure/576 nm emission) were used to observe the MitoTracker Orange CMTM Ros. An argon ions laser ray at 488 nm and the B-2 A filter (495 nm exposure/519 nm emission) was used to observe the DCF. Scanning was conducted with 25 optical series from the top to the bottom of the embryo with a step size of 0.45 μm to allow three-dimensional distribution analysis. General criteria for mt pattern definition were adopted on the basis of previous studies in mouse and human oocytes and embryos [13,15,45,46], as well as in oocytes of other species [47]. Thus, an homogeneous/even distribution of small mt aggregates (SA) throughout the cytoplasm was considered as an indication of low energy cytoplasmic condition. Heterogeneous/uneven distribution of small and/or large mt aggregates indicated metabolically active cytoplasm. In particular, the accumulation of active mitochondria in the peripheral cytoplasm (pericortical mt pattern) and/or around the nucleus (perinuclear and perinuclear/pericortical mt pattern, P/P) were considered as characteristic of healthy cytoplasmic condition. Embryos showing irregular distribution of large mt clusters unrelated to the specific cell compartments were classified as abnormal. To our knowledge, few studies have been reported to date on intracellular ROS localization and levels in mouse embryos [48,49] and no studies on cryopreserved mouse embryos have been reported.

### Quantification of Mitotracker Orange CMTM Ros and DCF fluorescence intensity

Measurements of fluorescence intensities were performed in embryos having either P/P or SA mt distribution pattern. Embryos showing abnormal mt distribution pattern were excluded from this analysis. In each individual embryo, the fluorescence intensity was measured at the equatorial plane, with the aid of the EZ-C1 Gold Version 3.70 software platform for Nikon C1 confocal microscope. A circle of an area (arbitrary value = 100 in diameter) was drawn in order to measure only the cytoplasmic area. Fluorescence intensity encountered within the programmed scan area was recorded and plotted against the conventional pixel unit scale (0–255). Quantification analysis was per formed only on embryos showing regular round shape, thus allowing the software set-up for quantification analysis in circle-shaped areas [39]. Parameters related to fluorescence intensity were maintained at constant values for all evaluations. In detail, images were taken under fixed scanning conditions with respect to laser energy, signal detection (gain) and pinhole size.

### Mitochondria/ROS colocalization analysis

Colocalization analysis of mitochondria and ROS was performed by using the EZ-C1 Gold Version 3.70 software. For each channel, the same threshold, set to the zero value, was used for the data set. Degree of colocalization was reported as a Pearson’s correlation coefficient quantifying the overlap degree between MitoTracker and DCDHF DA fluorescence signals [39,50].

### Statistical analysis

The rates of embryos showing different degrees of chromatin damage were compared between treated (SF or VF) and control groups and between treatments (FS vs VF) by the Chi-square test with the Yates correction for continuity. The rates of embryos showing different mt distribution pattern were compared between treated (SF or VF) and control groups and between treatments (FS vs VF), as a whole or as separated data according to chromatin damage level, whether of grade A, B or C, by the Chi-square test with the Yates correction for continuity. The Fisher’s exact test was used when a value of less than 5 was expected in any cell. For confocal quantification analysis of mt activity and intracellular ROS levels, the least-square means of the dependent variable (Mitotracker CMTM Ros and DCF fluorescence intensity) were calculated in examined samples and the statistical significance of the least-square means between treated and control groups was calculated by one-way ANOVA followed by Multiple Comparison Dunn’s or Dunnett’s methods (SigmaPlot software). For mt/ROS colocalization, mean values of Pearson’s correlation coefficient were compared between treated and control groups by one-way ANOVA followed by Multiple Comparison Dunn’s or Dunnett’s methods (SigmaPlot software). Differences with p < 0.05 were considered as statistically significant.

## Results

Three hundred and six mouse embryos, 69% of which were at the morula (M, n = 210) and 31% at the blastocyst (Bl, n = 96) stage, were randomly allocated as non-frozen controls (n = 88 embryos, 65 morulae and 23 blastocysts) or slowly frozen/controlled rate freezing (SF, n = 75 embryos, 49 morulae and 26 blastocysts) or vitrified/ultrarapid freezing (VF, n = 143 embryos, 96 morulae and 47 blastocysts) groups. Survival rates after freezing-thawing were at least 80% in both procedures (not significant, data not shown).

### Cryopreservation affects chromatin integrity at a greater extent at the morula than the blastocyst stage

**Figure 1:**
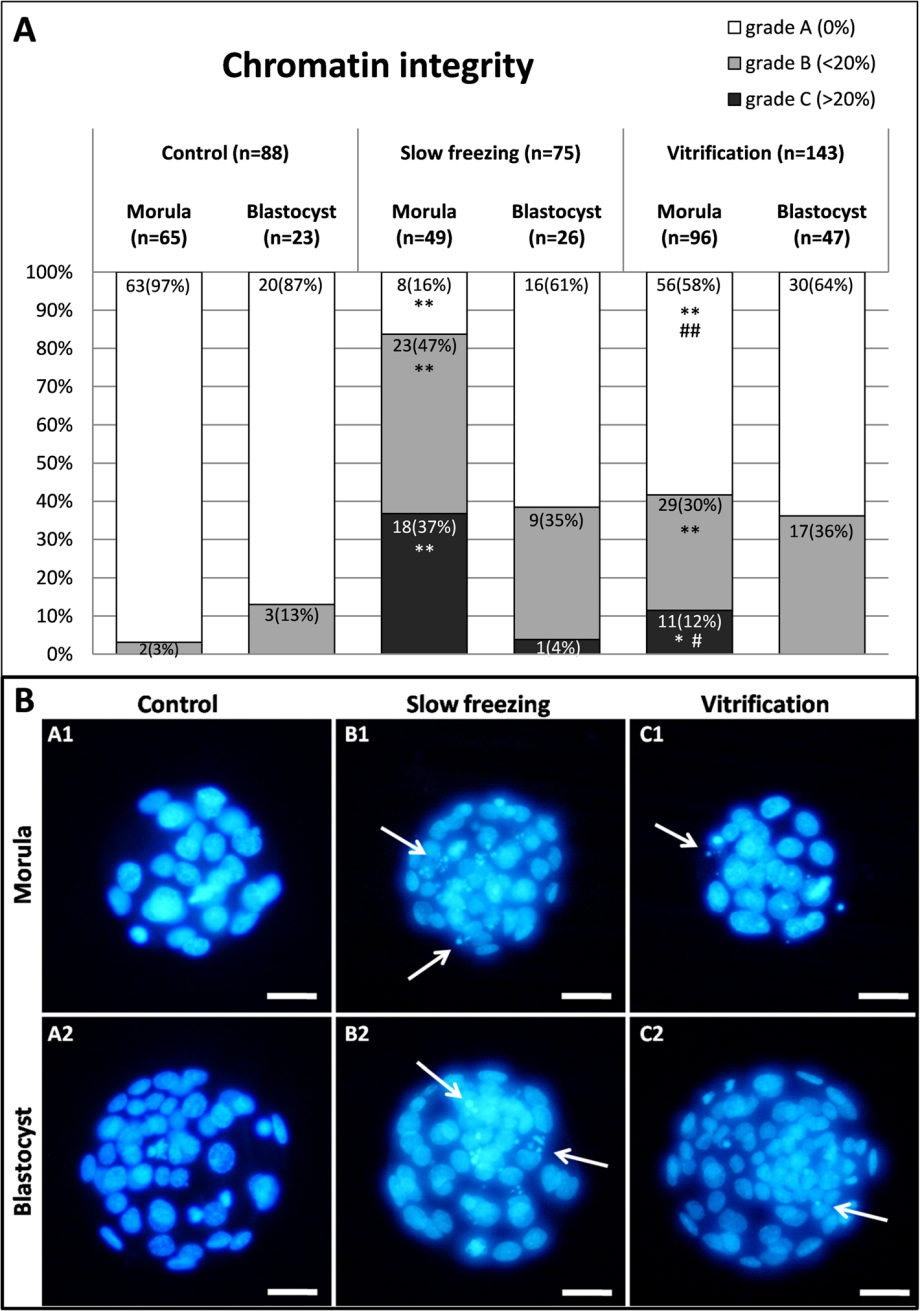
**Effects of slow freezing and vitrification on chromatin integrity of mouse embryos at the morula and blastocyst stage.** Panel **A**: percentages of embryos graded according to chromatin damage (for details and criteria see M&M) as grade A (no damage, white segments), grade B (slight damage, gray segments) or grade C (severe damage, black segments). Embryos were grouped according to their developmental stage, observed after slow freezing/thawing or vitrification/warming and compared with controls. Numbers of analyzed embryos per group are indicated on each histogram and segment. Chi square test with the Yates correction: comparisons slow freezing vs control and vitrification vs control: *P < 0.05; **P < 0.0001; comparisons slow freezing vs vitrification: # P < 0.001; ## P < 0.0001. Panel **B**: Representative photomicrographs of control grade A morula (A1) and control grade A blastocyst (A2), slow frozen grade C morula (B1) and slow frozen grade C blastocyst (B2), vitrified grade B morula (C1) and vitrified grade B blastocyst (C2) are shown. The nuclei of embryos were stained with Hoechst 33258. For each embryo, UV light images are shown. Arrows indicate signs of chromatin damage: white thin arrows indicate micronuclei and white thick arrows indicate lobulated nuclei. Scale bar represents 20 μm.

After both CP procedures, either SF or VF, chromatin damage was observed as formation of micronuclei or lobulated nuclei. In Figure 1 (Panel A), a percentage bar graph is reported in which embryos were graded as A, B and C, according to the described criteria (see Materials and Methods): grade A, embryos having all blastomeres with intact chromatin (Figure 1, Panel A, white segments), grade B, embryos having less than 20% blastomeres with damaged chromatin (gray segments) and grade C, embryos having more than 20% blastomeres with chromatin damage (black segments). In Figure 1 (Panel B), representative photomicrographs of embryos at the morula and blastocyst stage, and classified as grade A, B or C, are shown.

In the control group, the majority of embryos were of grade A (for M + Bl, 94%, 83/88), few embryos were of grade B (for M + Bl, 6%, 5/88) and no grade C embryos were found. In the SF group, the rates of grade A embryos were consistently reduced. In fact, significantly higher rates of grade B and C embryos were found (for M + Bl: 32/75, 43% vs 5/88, 6%, P < 0.0001 and 19/75, 25% vs 0/88, 0%, P < 0.0001 for B and C, respectively) compared with controls. In this group, 14 embryos (out of 19 grade C embryos) had less than 50% cells with chromatin damage and 5 embryos had more than 50% blastomeres with chromatin damage. In the VF group, the rates of grade B and grade C embryos also increased but at a lesser extent than in the SF group (for M + Bl: 46/143, 32% vs 5/88, 6%, P < 0.0001 and 11/143, 8% vs 0/88, 0%, P < 0.05 for B and C, respectively). In this group, all C embryos showed less than 50% blastomeres with chromatin damage. Furthermore, SF induced significantly higher chromatin damage than VF. In fact, even if the rates of grade B embryos did not change between the two methods (32/75, 43% vs 46/143, 32%, for SF and VF respectively, not significant) the rate of grade C embryos was significantly higher in the SF group than in the VF one (19/75, 25% vs 11/143, 8%, P < 0.001).

As for developmental stage-related effects, both CP procedures affected chromatin integrity of mouse pre-implantation embryos, with greater extent in morulae compared with blastocysts (Figure 1, Panel A). The rates of grade B and grade C morulae significantly increased after SF (23/49, 47% vs 2/65, 3%, P < 0.0001 and 18/49, 37% vs 0/65, 0%, P < 0.0001 for B and C morulae, respectively) and VF (29/96, 30% vs 2/65, 3%, P < 0.0001 and 11/96, 12% vs 0/65, 0%, P < 0.05 for B and C morulae, respectively) compared with controls. As well, even if the rates of grade B morulae did not change between the two methods (23/49, 47% vs 29/96, 30%, for SF and VF respectively, not significant), the rates of grade C morulae was significantly higher after SF than VF (18/49, 37% vs 11/96, 11%, P < 0.001). Conversely, chromatin integrity of embryos at the blastocyst stage was not affected by both CP methods, as no statistical differences were found among groups and comparable rates of grade A and grade B blastocysts were found, irrespectively of the CP treatment.

### Cryopreservation affects mitochondrial aggregation/distribution pattern at a greater extent at the morula than the blastocyst stage

**Figure 2:**
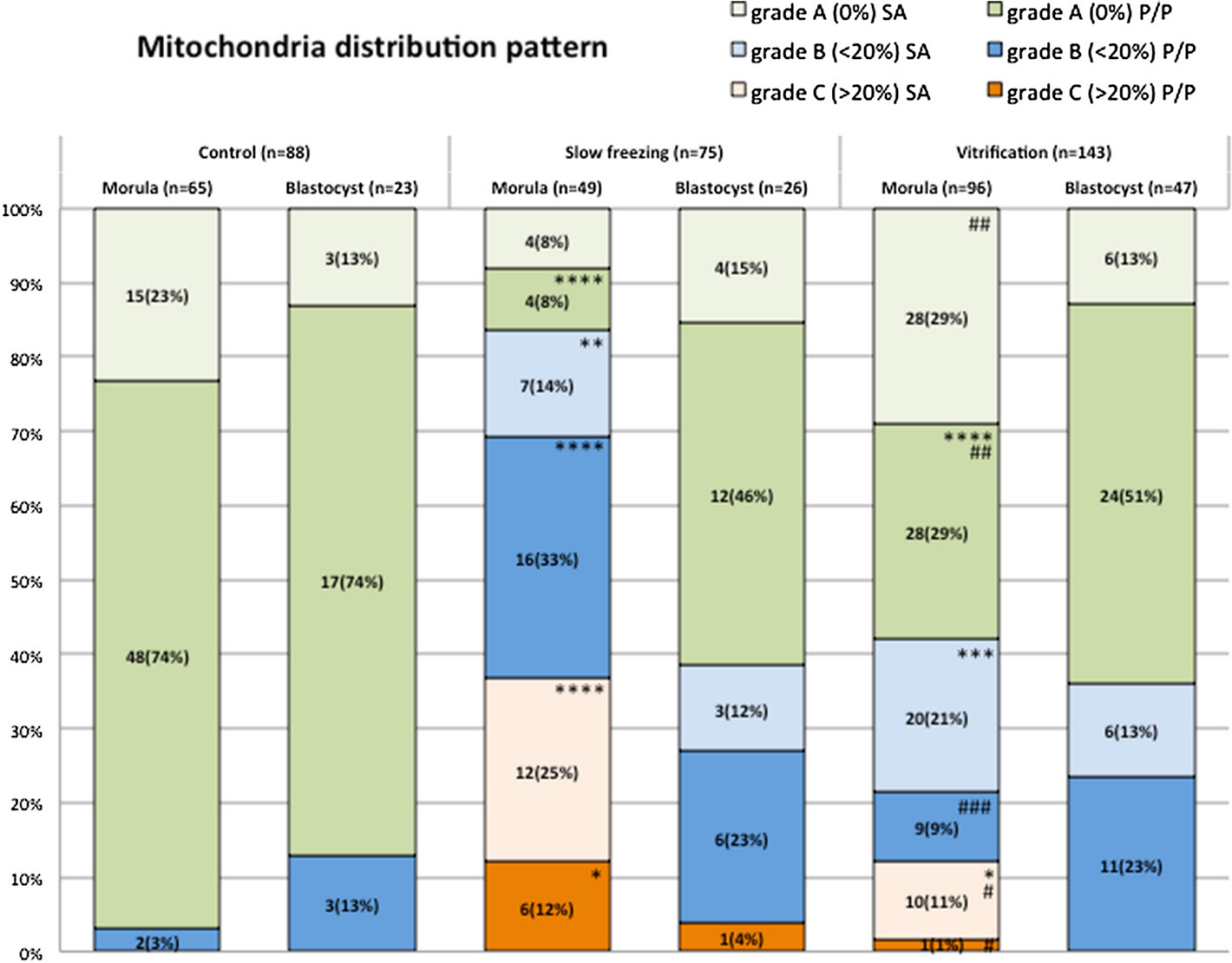
**Effects of slow freezing and vitrification on mitochondrial distribution pattern of mouse embryos at the morula and blastocyst stage.** Percentages of embryos graded according to chromatin damage as grade A, grade B or grade C, as in Figure 1, and further divided as having perinuclear/pericortical (P/P; apple-green, blue and brick-red, respectively) or homogeneous mt distribution pattern in small aggregates (SA; pale colours). Embryos were grouped according to their developmental stage, observed after slow freezing or vitrification and compared with controls. Numbers of analyzed embryos per group are indicated on each histogram and segment. Chi square test with the Yates correction: comparisons control vs slow freezing and control vs vitrification: *P < 0.05; **P < 0.01;***P < 0.001; ****P < 0.0001; comparisons slow freezing vs vitrification: #P < 0.05; ##P < 0.01; ###P < 0.001.

In both CP techniques, mt aggregation/distribution pattern in mouse pre-implantation embryos was found as either heterogeneous (pericortical/perinuclear distribution of mt clusters, P/P; Figure 2, grainy segments) or homogeneous (small mt aggregates, SA; smooth segments).

In the control group, the majority of embryos had P/P mt pattern. In fact, 80% (70/88) P/P and 20% SA embryos (18/88) were found. In both CP groups, the rates of embryos showing P/P pattern tended to decrease and corresponding increased rates of embryos showing the SA mt pattern were observed. In fact, significantly lower rate of embryos with P/P mt pattern was found in the SF (for M + Bl, grade A + B + C: 45/75, 60% vs 70/88, 79%, for SF vs controls, respectively; P < 0.05) and VF group (for M + Bl, grade A + B + C: 73/143, 51% vs 70/88, 79%, for VF vs controls, respectively; P < 0.0001) compared with controls. Furthermore, no differences were revealed between the two methods, as the comparisons between the rates of embryos with P/P pattern was not significantly different between SF and VF embryos (for M + Bl, grade A + B + C: 73/143, 51% vs 45/75, 60%, for VF vs SF, respectively; NS).

Cryopreservation methods differently affected mt pattern of mouse embryos according to their developmental stage, with greater extent in morulae compared with blastocysts. The rates of morulae with P/P pattern was significantly reduced after SF (26/49, 53% vs 50/65, 77%, for SF and controls respectively; P < 0.05) and VF (38/96, 40% vs 50/65, 77%, for VF and controls respectively; P < 0.0001) compared with controls. However, the comparison between the rates of heterogeneous morulae issuing from the two methods was not statistically significant (26/49, 53% vs 38/96, 40%, for SF and VF respectively, NS). In embryos at the blastocyst stage, mt aggregation/distribution pattern was not affected by both CP procedures, as no significant differences were found among groups. Comparable rates of blastocysts showing P/P mt pattern were found, irrespectively of the CP treatment (19/26, 73% vs 20/23, 87% for SF and controls, respectively, NS; 35/47, 74% vs 20/23, 87% for VF and controls, respectively, NS; 19/26, 73% vs 35/47, 74% for SF and VF, respectively; NS).

Further, the effects of CP on mt pattern varied according to embryo grade with heavier effects observed in grade C embryos. As overall data (including morulae and blastocysts), no differences were found between grade A and grade B embryos (133/193, 69% vs 47/83, 57% for grade A and B, respectively; NS) whereas significantly lower rates of embryos showing P/P mt pattern were found in grade C embryos compared with grade A (8/30, 36% vs 133/193, 69% for grade C and A, respectively; P < 0.0001) and grade B embryos (8/30, 36% vs 47/83, 57%, for grade C and B, respectively, P < 0.01). In grade A morulae, the P/P pattern was found at comparable rates (white grainy segments) in SF (4/8, 50%) and control groups (48/63, 76%; NS) whereas it was significantly reduced in the VF group (28/56, 50%) compared with controls (48/63, 76%; P < 0.01). In this group (grade A morulae), no statistical differences were found between methods (4/8 50%vs 28/56, 50% for SF vs VF grade A morulae, respectively; NS). In grade B morulae (gray grainy segments), no differences were found in the rates of embryos showing the P/P pattern between each CP method and controls (16/23, 69%, for FS 9/29, 31% for VF and 2/2, 100% for controls; NS). However, significantly lower rates were found in the VF group compared with the SF one (9/29, 31% vs 16/23 69% for VF vs SF, respectively; P < 0.05). In grade C morulae (black grainy segments), statistical comparisons for SF vs controls and VF vs controls were not feasible, and the comparison SF vs VF was not statistically significant. For any embryo category, no statistical differences were found between treatments groups in embryos at the blastocyst stage, as all comparisons were not statistically significant.

**Figure 3:**
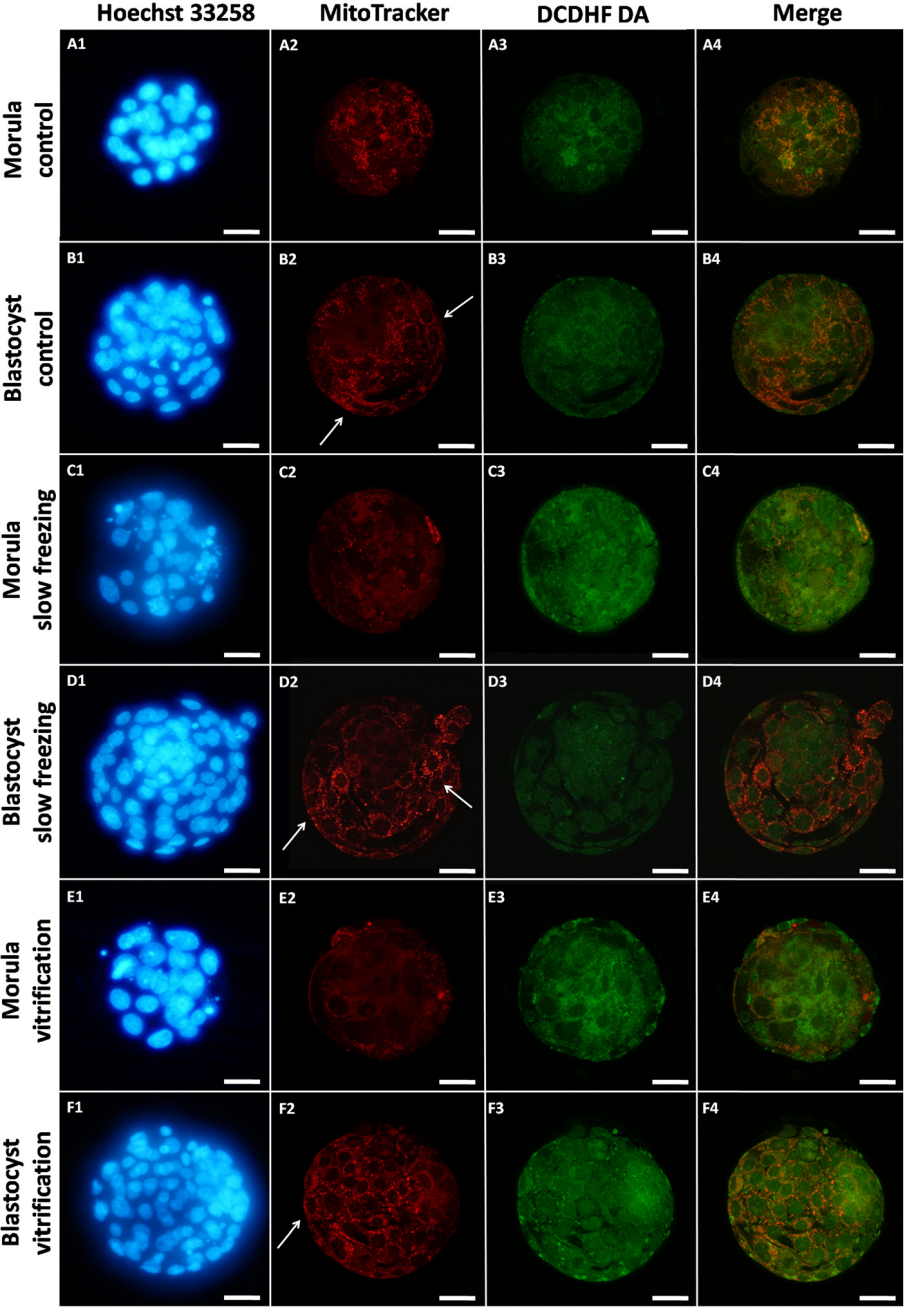
**Photomicrographs of frozen/thawed and vitrified/warmed mouse embryos at the morula and blastocyst stage of development as assessed for their nuclear chromatin and bioenergy/oxidative potential.** MitoTracker Orange, and DCDH FDA were used to label mitochondria and ROS, respectively. Nuclear chromatin was stained with Hoechst 33258. Representative photomicrographs showing mt distribution pattern and ROS intracellular localization in a control morula (row **A**) and a control blastocyst (row **B**) with P/P mt pattern, a SF morula with SA pattern (row **C**), a SF blastocyst with P/P pattern (row **D**), a VF morula with SA pattern (row **E**) and a VF blastocyst with P/P pattern (row **F**). In embryos at the blastocyst stage, a higher number of red fluorescent spots is evident on the trophoectoderma (white arrows) compared with the inner cell mass, indicating differences in mt number/cell between these two embryo lineages and higher mt/number and aggregate formation in the trophoectoderma compared with ICM. This feature can be observed in embryos of both groups, thus it was not influenced by cryopreservation. For each embryo, the corresponding epifluorescence images showing nuclear chromatin (line 1) and confocal images showing mt distribution pattern (line 2), ROS localization (line 3), mt/ROS merge (line 4) are shown. Scale bar represents 20 μm.

In Figure 3, representative photomicrographs of mouse morulae and blastocysts of control (rows A, B), slow freezing (rows C, D) and vitrification (rows E, F) groups and showing nuclear chromatin configuration (lane 1), and corresponding P/P or SA mt pattern (lane 2), intracellular ROS localization (lane 3) and mt/ROS merge (lane 4) are reported. In morulae and blastocysts with heterogeneous P/P mt pattern, in all blastomeres, there were detectable highly fluorescent signals of the mt-specific probe in the form of continuous rings around the nuclei and clusters of mitochondria at the cortex (Figure 3, A2, B2, D2, F2) which was reported in previous studies as indication of healthy embryos [51,52]. Due to reduced blastomere cytoplasmic size in embryos at the morula or blastocyst developmental stages, P/P mt clustering were almost overlapping. In morulae and blastocysts with homogeneous SA pattern, small mt aggregates diffused throughout the blastomere cytoplasm were observed (Figure 3, C2, E2). In addition, in embryos at the blastocyst stage, a higher number of red fluorescent spots was found on the trophoectoderm compared with the inner cell mass, indicating differences in mt number/cell between these two embryo lineages and higher mt/number and aggregate formation in the trophoectoderm compared with ICM (Figure 3, B2, D2, F2). This feature was observed in all groups (22% [5/23], 23% [6/26] and 32% [15/47] for control, SF and VF embryos: not significant) thus it was not influenced by CP procedures. Intracellular ROS localization did not vary upon CP and intracellular ROS appeared diffused throughout the cytoplasm of embryonic blastomeres at any stage of development (Figure 3, lane 3) apart areas/sites of mitochondria/ROS overlapping (Figure 3, lane 4).

### Cryopreservation alters quantitative bioenergy/redox parameters at a greater extent at the morula than the blastocyst stage

**Figure 4:**
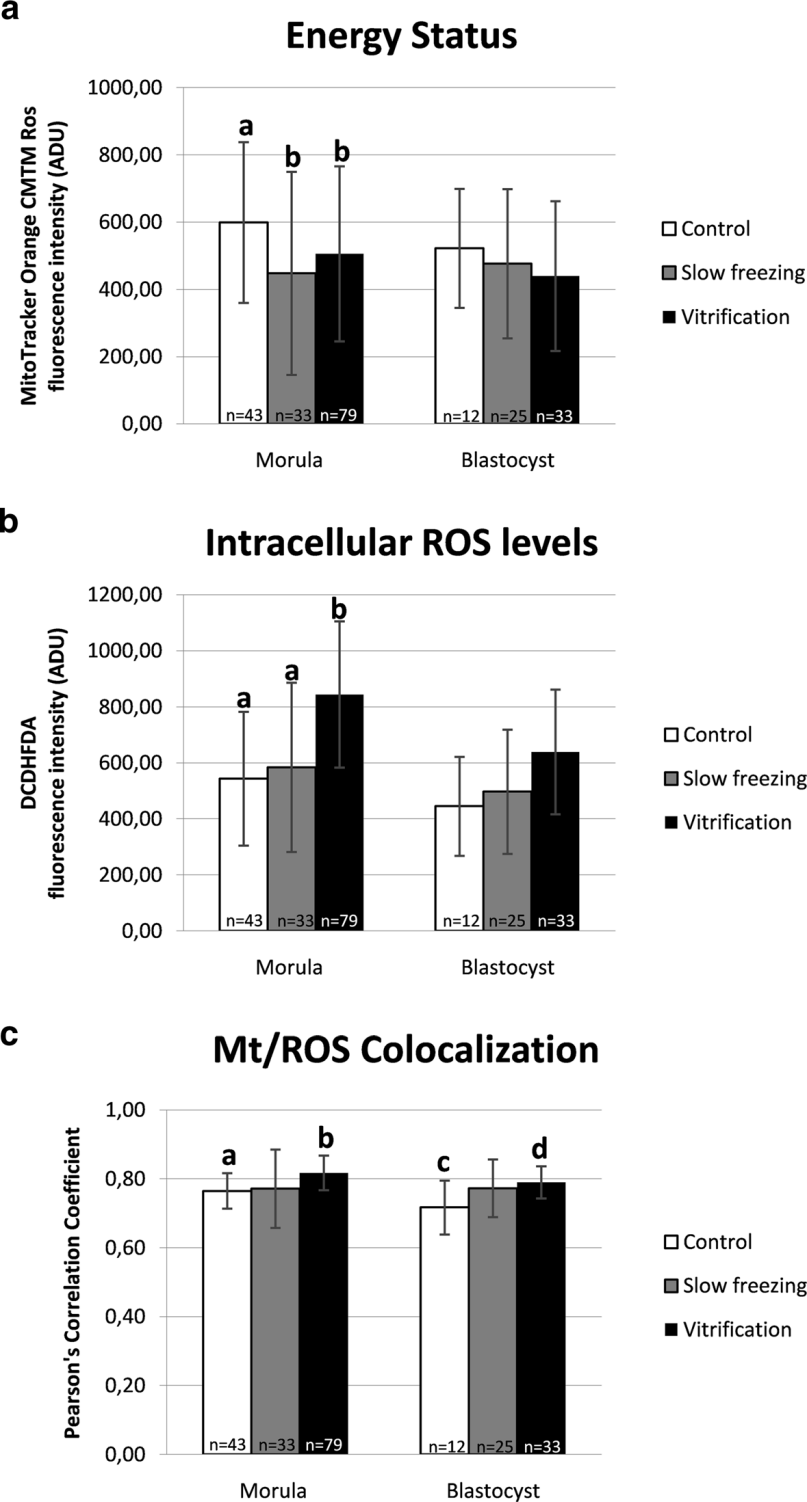
**Effects of slow freezing and vitrification on mitochondrial activity, intracellular ROS levels and mt/ROS colocalization in single mouse morulae and blastocysts.** In each group, energy status and ROS intracellular levels are expressed as mean ± SD of Mitotracker Orange CMTM Ros (Panel **a**) and DCF (Panel **b**) fluorescence intensity of individual embryos in arbitrary densitometric units (ADU) and mt/ROS colocalization is expressed as mean ± SD of Pearson’s correlation coefficient of individual embryos (Panel **c**). In embryos at the morula stage, energy status, expressing embryonic mt activity, was reduced by cryopreservation (P < 0.05) whereas in embryos at the blastocyst stage, it did not change. In embryos at the morula stage, ROS levels increased after vitrification (P < 0.05) whereas no changes were observed in embryos at the blastocyst stage. Vitrified morulae and blastocysts showed significantly higher Pearson’s correlation coefficient than controls (P < 0.05). Numbers of analyzed embryos per group are indicated on the bottom of each histogram. One-way ANOVA followed by Multiple Comparison Dunn’s method: comparisons among morula stage embryos: a,b P < 0.05; comparisons among blastocyst stage embryos: c,d P < 0.05.

**Figure 5:**
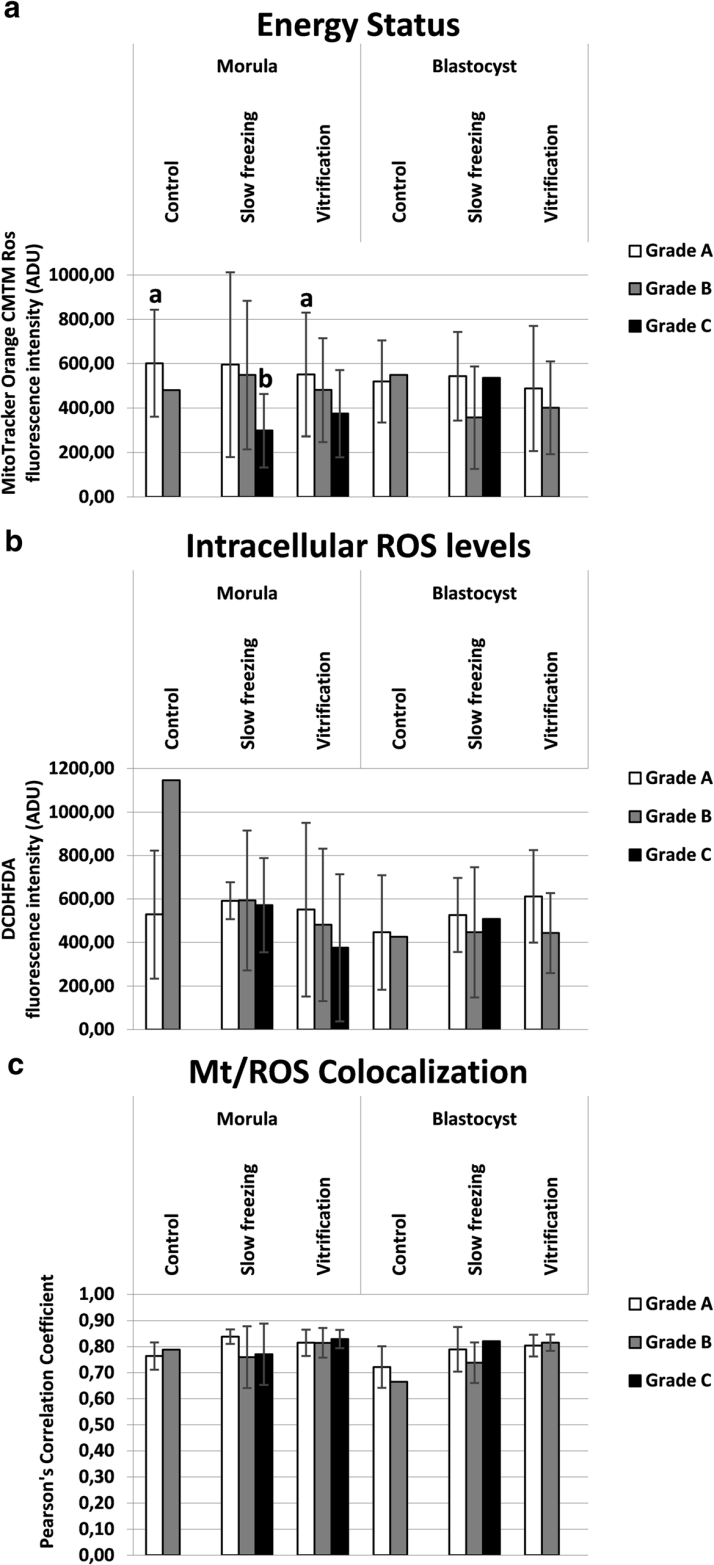
**Effects of slow freezing and vitrification on mitochondrial activity, intracellular ROS levels and mt/ROS colocalization in single mouse morulae and blastocysts, as related to embryo grade.** In each group, energy status (Panel **a**), intracellular ROS levels (Panel **b**) and mt/ROS colocalization (Panel **c**) are expressed as in Figure 4. In grade C SF morulae, energy status was significantly lower than in control grade A and in vitrified grade A morulae (P < 0.05). Numbers of analyzed embryos per group are indicated on the bottom of each histogram. One-way ANOVA followed by Multiple Comparison Dunn’s method: a,b P < 0.05.

**Figure 6:**
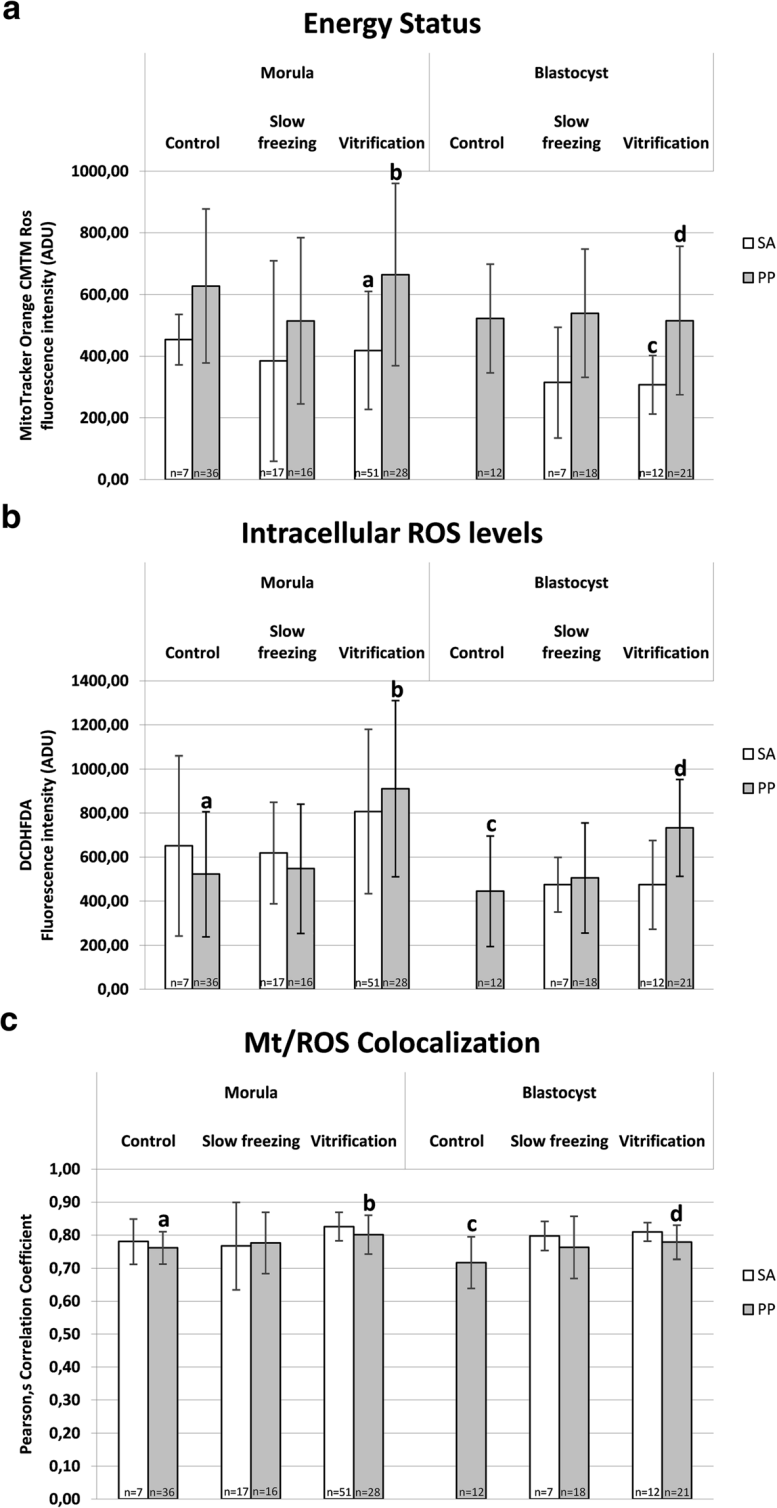
**Effects of slow freezing and vitrification on mitochondrial activity, intracellular ROS levels and mt/ROS colocalization in single mouse morulae and blastocysts, as related to mt pattern.** In each group, energy status (Panel **a**) intracellular ROS levels (Panel **b**) and mt/ROS colocalization (Panel **c**) are expressed as in Figure 4 and Figure 5. Vitrified embryos with P/P pattern had significantly higher energy status than their SA counterparts and significantly higher ROS levels and mt/ROS colocalization than P/P controls (P < 0.05). Numbers of analyzed embryos per group are indicated on the bottom of each histogram. One-way ANOVA followed by Multiple Comparison Dunn’s or Dunnett’s met hods: comparisons among morula stage embryos: a,b P < 0.05; comparisons among blastocyst stage embryos: c,d P < 0.05.

Mitochondrial activity, intracellular ROS levels and mitochondria/ROS colocalization were evaluated at the equatorial plane of embryos which were cryopreserved at the morula or blastocyst stage, having a round shape and thus allowing the confocal quantification software set-up in areas describing continuous surfaces [39,52]. Energy status, expressing embryonic mt activity, was significantly reduced upon application of both cryopreservation procedures in embryos at the morula stage, whereas it did not change in embryos at the blastocyst stage (Figure 4, Panel a; P < 0.05). Intracellular ROS levels were significantly increased in VF embryos at the morula stage (Figure 4, Panel b; P < 0.05) compared with controls whereas they did not change in blastocyst stage embryos. Moreover, ROS levels were significantly increased in VF compared with SF embryos. Mitochondria/ROS colocalization significantly increased in VF embryos compared with controls in both developmental stages (Figure 4, Panel c; P < 0.05). In Figure 5, quantification bioenergy/redox data were separated according to embryo grade. Due to the absence of grade C embryos in control morulae and blastocysts and in vitrified blastocysts, the column representing grade C embryos is lacking in these groups. In addition, only one grade B morula, one grade B blastocyst and one grade C blastocyst were examined, thus data of these samples are represented as single values and were not statistically analyzed. Statistical analysis revealed that energy status of SF grade C morulae was significantly lower than that of control grade A morulae (Figure 5, Panel a; P < 0.05). No differences were found for this parameter between treatments (SF vs controls, VF vs controls and SF vs VF) among grade A and among grade B embryos. No differences were found for intracellular ROS levels and mt/ROS colocalization between treatment and for any embryo grade. Figure 6 shows quantification bioenergy/redox data separated according to mt pattern. Due to the absence of control blastocyst with SA pattern with regular round shape, the corresponding column is lacking. Statistical analysis revealed that, for energy status, no significant differences between SF and controls for embryos having either P/P or SA pattern were observed, indicating that both embryo types contributed equally to energy status reduction observed after SF. Instead, energy status was significantly higher in VF embryos with P/P mt pattern compared with those having SA mt pattern, both at the morula and the blastocyst stage, indicating that, mt activity reduction in vitrified embryos is associated with the appearance of the SA pattern (Figure 6, Panel a; P < 0.05); 2) for intracellular ROS levels, significantly higher values were observed in VF embryos with P/P pattern compared with fresh ones with P/P pattern, both in morulae and blastocysts (Figure 6, Panel b; P < 0.05); 3) the same significances were revealed for mt/ROS colocalization (Figure 6, Panel c; P < 0.05). In embryos showing SA mt pattern, no differences were observed for any bioenergy/redox parameter between both treatments and controls.

## Discussion

While the impact of CP on the integrity of the oocyte/embryo plasma membrane, organelles, and spindle cytoskeleton have been the focus of most studies to date, the short- and long-term consequences of CP and cryoprotectants on the nuclear chromatin integrity has received much less attention [53]. In our study, both SF and VF significantly reduced the percentages of grade A embryos, thus impairing nuclear chromatin integrity. The extent of chromatin damage was higher after SF than VF, as only 32% of slow frozen embryos, but 60% of vitrified embryos had grade A (P < 0.0001). Moreover, chromatin damage induced by SF was much more evident in embryos at the morula than the blastocyst stage. In the group of morulae only 16% of the embryos, however in the blastocyst group 61% of the embryos had grade A (P < 0.001). Conversely, VF-induced chromatin damage was lower compared to SF (as assessed by prevailing appearance of grade B but no grade C embryos) and was equivalent in the morula and the blastocyst stage, as 58% of vitrified morulae and 64% of vitrified blastocysts had grade A (not significant).

Although not many studies have been published so far, our observations are in agreement with the results of previous studies. Even with different detection methods, damaging effects of CP procedures (SF and VF) on embryo chromatin integrity and function have been reported [34,54,55]. Isachenko et al. [21] found that the nuclear chromatin integrity rate after CP of pronuclear zygotes was a predictor of future embryo development and implantation. Tsang and Chow [56] reported significant reduction of DNA integrity in embryos after both SF and VF. Vutyavanich et al. [34] obtained significantly higher average number of nuclei in blastocysts derived from embryos vitrified at the 2-cell stage, and cultured in vitro, compared with those obtained after SF. Although the fact of morula being more cryosusceptible is well known [6], to our knowledge, our study is the first comparing developmental stage-specific effects of SF and VF on chromatin integrity of mouse morulae and blastocyst embryos.

Cryopreservation may have an effect on the structures of the embryo, but very mismatching data have been published up to now. Zhao et al. [15] observed that VF affected mitochondrial distribution, microtubule distribution and reduced the mitochondrial membrane potential in mouse 2-PN embryos, events which may have an influence on subsequent developmental viability of such embryos [15]. However, Noto et al. [14] found that freezing did not affect subcellular structures and the mitochondrial distribution appeared untouched after freezing – thawing. Their concl usion was that subcellular structures are rather resistant targets for cryoinjury [14]. Whole embryo confocal imaging allows qualitative and quantitative evaluation of several aspects of mt activity in single embryos, thus allowing to localize and quantify functional aberrations. Taking into consideration the mt aggregation and distribution pattern as a qualitative parameter of mt activity and cytoplasmic maturity, we found that both CP procedures, SF and VF, significantly reduced the rates of embryos showing the P/P mt pattern which indicative of cytoplasmic activity and maturity. In the group of grade B morulae, higher rates of SA patterns were found after VF than SF. It also came out that SF and VF affected mt pattern differently in relation to embryo developmental stage, with more relevant damage at the morula than the blastocyst stage. Furthermore, the effects of CP on mt pattern varied according to embryo grade with more serious effects on grade C embryos, thereby emphasizing the synergy between chromatin and mitochondrial pattern, which could correlate each other with loop mode. Experiences obtained with fresh morulae and blastocyst embryos indicated that: 1) mt localization was P/P in blastomeres of embryos at both the morula and blastocyst stage, 2) there were blastomeres with intense mt activity while others with lower activity, and 3) in embryos at the blastocyst stage, mt activity of trofoectodermal cells was more intense compared to cells located in the inner cell mass and blastomeres with strong mt activity were located nearby the blastocoelic cavity. Similarly, Van Blerkom [13] found that the maintenance of the blastocoel and its rapid recovery after the collapse and the hatching phase, are morphodynamics activities that require huge production of ATP from the trofoectodermal cells than the inner cell mass. Data obtained in mouse blastocysts show that 80% of ATP produced by the trofoectodermal cells and the number of mitochondria detected by confocal laser microscopy is very low in the inner cell mass [13]. Quantification analysis revealed a statistically significant reduction of MitoTracker fluorescence intensity in VF and SF morulae compared with their fresh counterparts, indicating significant reduction of mt activity at this stage of development after both CP procedures. In blastocysts, no significant effects were observed, even though data tended toward reduction after both procedures (Figure 4), as it was observed in a previous study from our unit [52]. Interestingly, energy status reduction observed in slow frozen morula-stage embryos cannot be associated with a specific level of chromatin damage, as slow frozen morulae did not differ for energy status among grade groups. Nevertheless, slow frozen grade C morulae showed significantly lower mt activity than grade A control and vitrified morulae (P < 0.05, Figure 5). Thus indicates that SF could induce a joint chromatin and energy status damage, however further studies are needed to confirm this hypothesis. In the VF group, energy status was also associated with P/P mt pattern, as morulae and blastocysts with P/P mt pattern showed higher mt activity than those with SA mt pattern (P < 0.05 for VF embryos, Figure 6). In embryos at the morula stage, a statistically significant increase of DCF fluorescence intensity, indicative of an excess production of ROS, was found after VF whereas ROS generation did not change after SF. ROS level variations were neither associated with embryo chromatin grade (Figure 5, NS) nor with mt pattern. Nevertheless, VF morulae and blastocysts with P/P mt pattern showed higher ROS staining than their corresponding controls (P < 0.05, Figure 6). This finding confirms previous observations from our unit [52] and allow us to lean more towards the hypothesis that the conditions used for the VF method may have stimulated oxidative activity and therefore cell viability rather than resulting in the onset of oxidative stress conditions. On the other hand, SF could have induced loss of embryo viability, as observed by reduced energy status and non increased ROS levels.

In the present study, mt/ROS colocalization was significantly increased by VF, both at the morula and blastocyst stage, but it was not increased by SF. These variations were neither associated with embryo chromatin grade (Figure 5, NS) nor with mt pattern (Figure 6, NS). To our knowledge, this is the first study reporting mt/ROS colocalization, objectively expressed as Pearson’s correlation coefficient, for the comparison between SF and VF in mouse preimplantation stage embryos.

## Conclusions

Cryopreservation affected chromatin integrity in mouse pre-implantation embryos at a greater extent at the morula than the blastocyst stage. Chromatin damage induced by SF was more relevant than that induced by VF (percentages of affected embryos and damage extent). Slow freezing and VF similarly affected mitochondrial aggregation/distribution pattern. Greater damage extent was observed at the morula stage and was associated with embryo grade, as lower rates of embryos showing P/P mt pattern were found in grade C compared with grade A and B embryos. Cryopreservation altered the quantitative bioenergy/redox parameters at a greater extent in morulae than in blastocysts. Effects induced by SF were not related to embryo grade or mt pattern, as affected embryos were of all grades and both mt patterns. Instead, effects induced by VF were related to mt pattern, as only embryos with SA mt pattern had reduced energy status. This study shows for the first time the joint assessment of chromatin damage and mitochondrial energy/redox potential in mouse embryos at the morula and blastocyst stage allowing the comparison of the effects of the two most commonly used cryopreservation procedures.
